# Open Retroperitoneal Repair for Complex Abdominal Aortic Aneurysms

**DOI:** 10.1055/s-0042-1748959

**Published:** 2022-11-01

**Authors:** Martin Hossack, Gregory Simpson, Penelope Shaw, Robert Fisher, Francesco Torella, John Brennan, Jonathan Smout

**Affiliations:** 1 Liverpool Vascular and Endovascular Service, Royal Liverpool and Broadgreen University Hospitals National Health Service Trust, Liverpool, United Kingdom; 2 Liverpool Centre for Cardiovascular Sciences, University of Liverpool, Liverpool, United Kingdom

**Keywords:** abdominal aortic aneurysm, complex, juxtarenal, suprarenal, retroperitoneal approach, open repair

## Abstract

**Background** Open surgical repair (OSR) of complex abdominal aortic aneurysms (CAAAs) can be challenging. We frequently utilize the retroperitoneal (RP) approach for such cases. We audited our outcomes with the aim of establishing the utility and safety of this approach.

**Methods** Retrospective analysis was performed of all patients undergoing OSR of an unruptured CAAA via a RP approach in our center over a 7-year period. Data on repairs via a transperitoneal (TP) approach were collected to provide context. Demographic, operative, radiological, and biochemical data were collected. The primary outcome measure was 30-day/inpatient mortality. Secondary outcomes included the need for reoperation, incidence of postoperative chest infection, acute kidney injury (AKI) and length of stay (LOS). All patients received aortic clamping above at least one main renal artery.

**Results** One hundred and three patients underwent OSR of an unruptured CAAA; 55 via a RP approach, 48 TP. The RP group demonstrated a more advanced pattern of disease with a larger median maximum diameter (65 vs. 61 mm, *p* = 0.013) and a more proximal extent. Consequently, the rate of supravisceral clamping was higher in RP repair (66 vs. 15%, *p* < 0.001). Despite this there were no differences in the observed early mortality (9.1 vs. 10%, NS); incidence of reoperation (10.9 vs. 12.5%, NS), chest infection (32.7 vs. 25%, NS), and AKI (52.7 vs. 45.8%, NS); or median LOS (10 vs. 12 days, NS) following RP and TP repair.

**Conclusion** OSR of CAAAs carries significant 30-day mortality. In patients unsuitable for fenestrated endovascular aortic repair or those desiring a durable long-term solution, OSR can be performed through the RP or TP approach. This study has demonstrated that in our unit RP repair facilitates treatment of more advanced AAA utilizing complex proximal clamp zones with similar perioperative morbidity and mortality compared with TP cases utilizing more distal clamping.

## Introduction

The management of abdominal aortic aneurysms (AAAs) adjacent to or involving the renal arteries (RAs) poses a challenge to vascular specialists. The lack of a suitable infrarenal neck compromises standard endovascular aneurysm repair (EVAR), while open surgical repair (OSR) is complicated by insufficient space for infrarenal clamping, making more proximal clamp positions mandatory. Yet, these complex AAAs (CAAAs) make up 15% of all aneurysms needing treatment. 1

The widespread enthusiasm for and acceptance of EVAR to treat AAA has led to a decrease in OSR. 2 3 EVAR has been shown to offer improved early postoperative mortality 4 and morbidity and shorter length of stay (LOS). 5 Long-term outcomes, however, favor OSR, with significantly higher rates of reintervention and aortic rupture reported following EVAR. 6 7 For patients with CAAAs, in whom standard EVAR is not feasible, the endovascular options include fenestrated EVAR (FEVAR), branched EVAR, or chimney-EVAR (ChEVAR). ChEVAR has been shown to be useful, especially in urgent situations, where it would be inappropriate to delay intervention to permit manufacture of a custom-made device. However, outcomes have been shown to be inferior to FEVAR. 8 9 In certain patients OSR remains the preferred treatment choice. The preference for OSR can relate to unfavorable anatomy for endovascular repair, patient concerns over the requirement for lifelong follow-up and radiation exposure, and the need for secondary intervention.

The midline transperitoneal (TP) incision continues to be used for most open aortic surgery. 10 It provides excellent access to the infrarenal aorta, iliac and visceral arteries. Its utility in general and vascular surgery makes it most familiar to many vascular surgeons. However, access to the visceral aortic segment through this approach is limited and requires extensive dissection, as in medial visceral rotation.

Dubost et al 11 utilized a thoracoabdominal incision and retroperitoneal (RP) approach in the first described surgical AAA repair in 1952. This approach to the aorta was subsequently utilized 12 and validated 13 for surgery on the *infrarenal* aorta in the 1960s. Williams et al modified the original technique with a more posterior incision, facilitating access to the *suprarenal* aorta. 14 15 This technique has been enhanced over time, with modifications of the incision used to tailor access to the desired proximal aortic segment. 16

Following centralization of vascular services and formation of Liverpool Vascular and Endovascular Service, there has been a pooling of expertise and experience. As a tertiary referral center, we have been treating increasing numbers of CAAAs, by both endovascular and open approaches. We frequently utilize the RP approach for OSR of CAAAs as we find it provides better access to the visceral aortic segment. There is also evidence that postoperative complications such as ileus and pneumonia, and length of intensive care unit stay are lower following AAA repair via a RP rather than TP approach. 17 18 19 20 Herein, we present a single-center experience of RP repair for unruptured CAAA, and repairs via a TP approach for context.

## Methods

From September 2012 to September 2019, consecutive patients undergoing OSR for an unruptured CAAA via a RP approach in our institution were retrospectively evaluated. Data on complex repairs via a TP approach over the same period were collected to provide context. The study was classified as a service review and registered with the hospital clinical effectiveness team (study number AC04155). Formal ethical approval was waived.

CAAA was defined as any aortic aneurysm requiring a cross clamp above at least one main RA. Patients with ruptured aneurysms were excluded. In our institution, the approach for repair is determined largely by aneurysm extent and surgeon's preference, with RP repair generally reserved for more proximal AAAs.

Demographic, pre-, intra-, and postoperative data, including clinical presentation, clamp level, and serum creatinine (SCr) results were collected. Preoperative contrast-enhanced computerized tomography (CT) scans were scrutinized for maximum aneurysm diameter and proximal aneurysm extent.

The primary outcome measure was 30-day or inpatient mortality. Secondary outcomes included the incidence of postoperative chest infection, acute kidney injury (AKI), reoperation, and LOS. Postoperative chest infection was defined as having occurred if the patient was commenced on antibiotics with a suitable respiratory indication documented. AKI presence and staging was defined according to local biochemistry laboratory criteria based on the Kidney Disease: Improving Global Outcomes classification. 21 The highest postoperative SCr was compared with the baseline preoperative value, and AKI stage defined as: Stage 1, increase 1.5–1.9 × baseline; Stage 2, increase 2–2.9 × baseline; Stage 3, increase in ≥ 3 × baseline OR initiation of renal replacement therapy.

Aneurysm classification was defined according to extent on CT: aneurysms with an outer-to-outer aortic diameter at the level of the lowermost RA of <30 mm were defined as “juxtarenal” (JRAAA); aneurysms with an outer-to-outer aortic diameter at the level of the lowermost RA > 30 mm and at the level of the celiac axis of <30 mm, “suprarenal”; and aneurysms with an outer-to-outer aortic diameter at the level of the celiac axis of >30 mm, “Type IV thoracoabdominal” aneurysm (TAAA).

All RP cases were evaluated for suitable proximal clamp position if performed via TP incision, and the subgroup of JRAAAs repaired via a RP approach were also assessed for hypothetical suitability for FEVAR, independently by authors R.F. and M.H., with disagreement resolved by discussion. Suitable neck morphology for FEVAR was defined according to the Instructions For Use for the Zenith Fenestrated Endovascular graft (Cook Medical, Brisbane, Australia): a non-aneurysmal infrarenal aortic segment proximal to the aneurysm with a length of at least 4 mm; a diameter measured outer wall to outer of no greater than 31 mm and no less than 19 mm; an angle less than 45 degrees relative to the long axis of the aneurysm; and an angle less than 45 degrees relative to the axis of the suprarenal aorta. The number of fenestrations required was calculated by counting the visceral branches covered in 20 mm of parallel aortic neck including the most proximal branch if this was partially covered. Hypothetical suitable clamp position via a TP approach was defined as 4 mm length of parallel aorta proximal to a cuff of non-aneurysmal aorta suitable for a sutured anastomosis in the authors' experience.

### Statistical Analysis

IBM SPSS Statistics for Windows, version 24 (IBM Corp., Armonk, NY) was used for statistical analysis. Univariate analysis was performed using the chi-square test or Fisher's exact test for categorical data. The *t* -test was used for continuous data with normal distribution and the Mann–Whitney *U* test for skewed data. For all statistical analyses, *p* < 0.05 was considered significant.

## Results

### Study Population

During the study period 55 patients underwent RP repair of an unruptured CAAA, while 48 underwent repair via a TP approach. Demographic and clinical data are summarized in Table 1 .

**Table 1:** Study population and operative data

Variable		TP ( *n* = 48)	RP ( *n* = 55)	Mean difference (95% CI)	*p* -Value
Male		39 (81)	44 (80)		0.873
Age, y mean ± SD		72.6 ± 7.0	69.8 ± 6.7	2.77 (0.9, 5.4)	0.043 a
ASA grade	1 or 2	19 (40)	18 (33)		0.420
	3	28 (60)	37 (67)		
Comorbidity	Diabetes	8 (20) *n* = 40	9 (18) *n* = 50		0.810
	Hypertension	19 (48) *n* = 40	36 (71) *n* = 51		0.025 a
	CLD	13 (33) *n* = 40	14 (27) *n* = 51		0.601
	IHD	10 (26) *n* = 39	23 (45) *n* = 51		0.058
	CKD	2 (5) *n* = 40	4 (8) *n* = 51		0.691
	CVD	3 (8) *n* = 40	6 (12) *n* = 51		0.726
Smoking	Current	11 (23)	21 (38)		0.109
	Ex or never	36 (76.6)	34 (61.8)		
Medication	Statin	47 (94) *n* = 47	44 (86) *n* = 51		0.322
	SAPT	28 (62.2) *n* = 45	33 (62.3) *n* = 53		0.997
	DAPT	2 (4.4) *n* = 45	3 (5.7) *n* = 53		0.785
	Anticoagulant	4 (9.1) *n* = 44	3 (5.7) *n* = 53		0.516
	Beta-blocker	14 (30) *n* = 47	17 (36) *n* = 47		0.510
	ACEi	13 (28) *n* = 47	16 (35) *n* = 46		0.458
BMI, kg/m ^2^ mean ± SD		26.6 ± 3.4 *n* = 36	26.5 ± 4.0 *n* = 51	0.1 (–1.5, 1.7)	0.904
Maximum AAA diameter, mm median (IQR)		61 (57.3, 67.5)	65 (60, 72)		0.013 a
Aortic status	Asymptomatic	48 (100)	52 (94.5)		0.246
	Symptomatic	0 (0)	3 (5.5)		
Previous aortic intervention	None	41 (85.4)	45 (83.3)		0.624
	EVAR	4 (8.3)	1 (1.9)		
	OSR	3 (6.3)	7 (13)		
	Unspecified	0	1 (1.9)		
CT extent	Suprarenal/Type IV TAAA	10 (21)	32 (58.2)		< 0.001 a
	Juxtarenal	38 (79)	23 (41.8)		
Clamp level	Supravisceral	7 (15)	36 (66)		< 0.001 a
	Above ≥1 renal artery only	41 (85)	19 (35)		

Abbreviations: AAA, abdominal aortic aneurysm; ACEi, angiotensin-converting enzyme inhibitor; ASA, American Society of Anesthesiologists; BMI, body mass index; CI, confidence interval; CKD, chronic kidney disease; CLD, chronic lung disease; CT, computed tomography; CVD, cerebrovascular disease; DAPT, dual antiplatelet therapy; EVAR, endovascular aneurysm repair; IHD, ischemic heart disease; IQR, interquartile range; OSR, open surgical repair; RP, retroperitoneal; SAPT, single antiplatelet therapy; SD, standard deviation; TAAA, thoracoabdominal aortic aneurysm; TP, transperitoneal.

Note: *n* , given when data not available for all patients, indicates number of patients from whom data available.

a Significant difference at 95% level of certainty.

The RP cohort presented with a more complex pattern of disease than the TP cohort, including larger maximum diameter and a more proximal extent on CT. Consequently, the rate of supravisceral (supramesenteric or supraceliac) clamping was significantly higher in the RP cohort (66% vs. 15%, *p* < 0.001).

### Outcomes

There were no significant differences in primary or secondary outcomes between the TP or RP groups ( Table 2 ).

**Table 2:** Outcomes by approach

Outcome		TP ( *n* = 48)	RP ( *n* = 55)	Mean difference (95% CI)	*p* -Value
Mortality (%)		5 (10.4)	5 (9.1)	1.3 (–10.2, 12.8)	1.000
Length of stay (d) median (IQR)		12 (8, 16)	10.5 (7, 16)		0.705
Maximum postop creatinine, µmol/L median (IQR)		127 (102.3, 175.8)	136 (99.8, 218)		0.599
Change in creatinine, µmol/L median (IQR)		31.5 (13.5, 92.5)	43.5 (12.8, 118.5)		0.524
AKI (%)	All	22 (45.8)	29 (52.7)	6.9 (–12.4, 26.2)	0.485
	Stage 1	9 (18.8)	14 (25.5)		
	Stage 2	11 (22.9)	11 (20)		
	Stage 3	2 (4.2)	4 (7.3)		
Reoperation (%)		6 (12.5)	6 (10.9)	1.6 (–4.8, 8.0)	0.802
HAP (%)		12 (25)	18 (32.7)	7.7 (–9.7, 25.1)	0.389

Abbreviations: AKI, acute kidney injury; CI, confidence interval; HAP, hospital-acquired pneumonia; IQR, interquartile range; RP, retroperitoneal; TP, transperitoneal.

Analysis of outcomes by clamp level rather than approach was also performed, demonstrating a significantly worse early mortality following supraceliac clamping ( Table 3 ).

**Table 3:** Outcomes by clamp level

Outcome	Clamp below celiac axis ( *n* = 68)	Supraceliac clamp ( *n* = 35)	Mean difference (95% CI)	*p* -Value
Mortality (%)	3 (4.4)	7 (20)	15.6 (1.5, 29.7)	0.029 a
Change in creatinine, µmol/L median (IQR)	32.5 (13.5, 83)	64 (13.5, 142.75)		0.118
AKI (%)	31 (45.6)	20 (57.1)	11.5 (–8.7, 31.7)	0.303
Reoperation (%)	5 (7.4)	7 (20)	12.6 (–2.0, 27.2)	0.101
HAP (%)	20 (29.4)	10 (28.6)	0.8 (–17.7, 19.3)	0.929

Abbreviations: AKI, acute kidney injury; CI, confidence interval; HAP, hospital-acquired pneumonia; IQR, interquartile range.

a Significant difference at 95% level of certainty.

#### Mortality

Ten patients died within 30 days of, or during initial hospitalization for, open CAAA repair ( Table 4 ). The median interval from operation to death was 10.5 days (range 0–53).

**Table 4:** Characteristics of patients suffering early mortality

Approach	Age (y)	Gender	Indication for repair	Diameter (mm)	CT extent	Clamp level	Interval to death (d)	Cause of death
RP	79	F	Asymptomatic	63	Suprarenal	SC	53	Pneumonia
RP	81	M	Asymptomatic	94	Suprarenal	SC	12	Stroke
RP	74	M	Asymptomatic	60	Suprarenal	SC	4	Bowel ischemia
RP	74	M	Asymptomatic	78	Juxtarenal	SC	14	Bowel ischemia
RP	60	F	Symptomatic	61	Suprarenal	SC	9	Hemorrhage
TP	74	M	Asymptomatic	58	Juxtarenal	SR	1	MOF
TP	79	M	Previous EVAR	120	Juxtarenal	SR	19	Bowel ischemia
TP	78	M	Asymptomatic	95	TAAA	SC	1	Myocardial infarction
TP	77	M	Asymptomatic	64	Juxtarenal	SR	32	Pneumonia
TP	71	M	Previous EVAR	83	Juxtarenal	SC	0	Myocardial infarction

Abbreviations: CT, computed tomography; EVAR, endovascular aneurysm repair; F, female; M, male; MOF, multiorgan failure; RP, retroperitoneal; SC, supraceliac; SR, suprarenal;TAAA, thoracoabdominal aortic aneurysm; TP, transperitoneal.

#### Reoperation

A total of 12 patients (11.7%) required reoperation (6 RP, 6 TP), of which 4 required two separate procedures making a total of 16 reoperations ( Table 5 ). Seven patients (6.8%) had to return for bleeding on a median postoperative day 1. Three patients (2.9%) returned to theater for bowel ischemia on median day 3 postop. One patient returned to theater for each of limb ischemia (1%), renal graft occlusion (1%), adhesional bowel obstruction (1%), insertion of nasogastric feeding tube (1%), surgical tracheostomy (1%), and endoscopic ultrasound with insertion of pancreatic stent for iatrogenic fistula (1%), on day 0, 1, 12, 10, 23, and 99, respectively.

**Table 5:** Indications for early reoperation

Approach	Indication 1	Interval 1 (d)	Indication 2	Interval 2 (d)	Survival to discharge
RP	Tracheostomy	23			Y
RP	Bleeding	0			Y
RP	Ischemic bowel	3			N
RP	Limb ischemia	0	Bleeding	9	N
RP	Bleeding	1			Y
RP	Bleeding	1	Occluded renal graft	1	Y
TP	Bleeding	0			Y
TP	Ischemic bowel	18			N
TP	Bleeding	2			Y
TP	Ischemic bowel	2	Bleeding	16	Y
TP	Adhesional obstruction	12	Pancreatic fistula	99	Y
TP	Insertion of NG	10			Y

Abbreviations: NG, nasogastric; RP, retroperitoneal; TP, transperitoneal.

### Assessment of Suitability for Alternative Treatment Options

All 55 CAAAs undergoing RP repair were assessed for hypothetical proximal clamp position if repaired via a TP approach. Using a TP approach 48/55 (87.3%) patients would have required supraceliac proximal clamp position compared with 31/55 (56.4%) via the performed RP approach ( *p* =0.002).

Of all 55 CAAAs repaired via RP approach 23 patients with a JRAAA extent on preoperative CT were assessed for suitability for FEVAR. Fourteen patients (60.1%) were considered suitable for FEVAR with either 3 ( *n* = 3) or 4 fenestrations ( *n* = 10). Nine patients (39.1%) were considered unsuitable for FEVAR, for infrarenal angulation >45 degrees (6), calcified aortic narrowing (1), previous EVAR with suprarenal fixation (1), and a common superior mesenteric artery (SMA)/celiac origin (1).

## Discussion

We present the early outcomes from 7 years' experience of OSR for unruptured CAAA via a RP approach in a single vascular tertiary referral center. We provide comparable baseline and outcome measures in TP repairs for context. Our results indicate equivalent early mortality, rate of reoperation, and incidence of AKI and chest infection, despite the RP repairs representing a cohort with more advanced aneurysmal disease on preoperative CT, with a larger maximum aortic diameter and more proximal aneurysm extent. We also demonstrate that the use of the RP approach allowed placement of a suprarenal or supramesenteric proximal clamp, in certain patients who would otherwise have required a supraceliac clamp using the TP approach.

Varkevisser et al 22 examined outcomes following JRAAA repair using data extracted from the American College of Surgeons National Surgical Quality Improvement Program. They found supraceliac clamping was associated with significantly higher 30-day mortality (odds ratio [OR] 3.4), reoperation (OR 2.4), and major complication (OR 2.2) compared with suprarenal clamping on multivariate analysis with adjustment for renal and visceral revascularizations (and therefore disease extent). Assessment of our patients' outcomes by clamp location rather than approach illustrated a significantly higher early mortality associated with supraceliac clamping. Keeping the proximal clamp as distal as possible is clearly of benefit. 23

We found no difference in the incidence of postoperative chest infection or LOS following RP and TP repair. Several other studies have compared the perioperative outcomes following AAA repair via a RP or TP approach. In agreement to our study, Sieunarine et al 24 found no benefit of the RP approach for chest infection or LOS. Other studies have shown no difference in postoperative respiratory complications but shorter LOS. 17 25

The largest series comparing RP ( *n* = 347) with TP ( *n* = 788) repair for AAA looked at only infrarenal and juxtarenal AAAs, a less advanced disease cohort than in this study. They noted that the RP approach was associated with a higher incidence of chest infection and a longer LOS than TP. 26

We did not find a significant difference in the incidence of AKI or change in SCr between the RP and TP cohorts despite a significantly higher incidence of supravisceral clamping in the RP repairs. Furthermore, regrouping of our data by clamp position confirms the lack of a significant difference. Sarac et al 27 previously demonstrated that supravisceral clamping was associated with a greater incidence of renal dysfunction, despite shorter clamp and therefore renal ischemia times. In contrast, and in common with the present study, Shortell et al, 28 Knott et al. 29 and Deery et al 10 found no such difference. All patients in our study received a proximal clamp above at least one main RA, but we have made no distinction between interrenal (below one main RA) and suprarenal (above both main RAs). Varkevisser et al 22 observed no difference in postoperative outcomes, including renal dysfunction, between clamping above one versus above both main RAs in repair of JRAAA, likely making this omission insignificant. Jongkind et al 1 meta-analyzed the results of 21 nonrandomized studies investigating the outcomes following JRAAA repair (1,256 patients) up to December 2008. No definite conclusions could be drawn about the superiority of either approach in this cohort of patients.

Chaufour et al 30 reported on OSR of 315 JRAAAs across 5 centers in France over a 10-year period from 2005, 67.3% via a RP approach. They found an early mortality of only 0.9% with their series representing a less advanced burden of disease, having a lower median maximal diameter (59.7 mm) and a very low proportion of supraceliac clamping (3.5%). Deery et al similarly observed a comparatively low early mortality (3.6%) following OSR (40% RP) of CAAA ( *n* = 443). The rate of supraceliac clamping was only 23%, and neither of these studies reported explicitly on the extent of disease, making direct comparison with our data difficult. A contemporary analysis of 957 patients undergoing elective OSR of a CAAA in the United States has revealed a 30-day mortality of 6.8%. This lower mortality reflects a greater proportion of JRAAA (63%) than in our RP cohort (42%), in whom the mortality was found to be lower than suprarenal AAA (SRAAA; 4.6% vs. 9.5%) and Type IV TAAA (14.7%). 31 The early mortality rate in our institution via both the RP (9.1%) and TP (10.4%) approach compares favorably with the U.K. average, according to both the 2017 (18.4%) 32 and 2019 (14.7%) 33 National Vascular Registry annual reports. Similarly, our rates of reoperation following TP and RP repair (12.5 and 10.9%) are lower than those reported nationally in 2017 (17.1%) 32 and 2019 (18.9%). 33

In our series, 3 patients underwent RP repair for a symptomatic AAA, of whom 1 died. Exclusion of symptomatic cases would improve the early mortality further.

The alternative to OSR repair for CAAA is FEVAR, in anatomically suitable cases. Early mortality following FEVAR for CAAA in the U.K. was 3.9 32 and 2.1% 33 in 2017 and 2019. In our series, 39.1% of JRAAAs were not suitable for FEVAR, and so had no alternative for treatment of their aneurysm. The remaining 60.1% were considered suitable for FEVAR, with 3 or 4 fenestrations. Data from the GLOBALSTAR database highlighted a trend for higher early mortality following more complex FEVAR—9.4% in patients having grafts incorporating the celiac trunk. 34 In our series, no JRAAA patient suitable for FEVAR but undergoing OSR suffered early mortality.

Interestingly, the TP cohort contained a higher proportion of TAAA according to our definition (7 vs. 1). Of these, only 3 underwent supraceliac clamping, while 4 had suprarenal clamps. It is possible that a suboptimal site for proximal anastomosis was “accepted” in these patients to simplify the procedure and reduce perioperative risk at the expense of long-term freedom from proximal aneurysmal dilatation. We believe one of the main advantages of the RP approach is to allow safe access to healthy proximal aortic tissue for a sutured anastomosis.

### Limitations

*Definitions of aneurysm extent* : There are no universally accepted definitions for aneurysms involving the visceral segment of the aorta. The Society for Vascular Surgery defined JRAAAs as those abutting but not involving the RAs, but did not discriminate between suprarenal and Type IV TAAAs. 35 Similarly, the European Society for Vascular Surgery defined JRAAAs as extending up to but not involving the RAs, that is, a *short neck* < 10 mm. Whereas they define SRAAAs, as extending up to the SMA, that is, *no neck* . They do not, however, offer a distinction between SRAAA and Type IV TAAA. 36 Type IV TAAAs were defined by Crawford et al 37 as extending from the 12th intercostal space to the iliac bifurcation, involving the visceral aortic segment and the origins of the renal, superior mesenteric, and celiac arteries. They are frequently grouped together with JRAAAs and SRAAAs, 38 and have been so in this series. Comparison with other series is limited by a lack of consensus for definition of proximal aneurysm extent.

*Retrospective study limitations* : Due to the retrospective nature of this study, we were not able accurately to record the presence or duration of postoperative ileus, for which the RP approach has been shown to be particularly beneficial. 17 18 39 40 Reduction in postoperative ileus associated with RP repair is thought to be due to the avoidance of opening the peritoneal cavity and handling of the intra-abdominal contents with consequent edema. In our study, the majority of patients treated with a RP approach required supraceliac clamping, temporarily interrupting the arterial supply to the abdominal viscera. Whether in this context the RP approach has the same benefit for ileus has not been investigated to our knowledge. The majority of infrarenal AAA repairs in our center are performed using a TP route with the RP approach reserved for patients in whom access to the visceral aortic segment is needed.

The retrospective nature of this study carries inherent limitations regarding data available for analysis. We relied upon prospectively completed electronic databases for much of the clinical data and hard endpoints. Therefore, specific intraoperative details were not available. For instance, it is possible in some cases that a distally placed clamp was resited proximally for control of bleeding. Furthermore, we have not collected data on intraoperative cold renal perfusion, which is implemented in our center for most CAAA repairs involving RA revascularizations, and may offer renal protection. No data on the occurrence of postoperative wound complications was collected. DeCarlo et al 41 found the RP approach to have a lower risk of laparotomy complications, such as hernia and small bowel obstruction, whereas Ma et al 20 found hernia and chronic wound pain to be higher post-RP repair.

*Nonblinded study* : Blinding for assessment of preoperative CT scans was not performed, which introduces the possibility of observation bias regarding hypothetical TP clamp position and suitability for FEVAR. Furthermore, we did not assess the suitability of the femoral vessels for access, which would have affected patient suitability for FEVAR.

*No specific criteria for approach* : Finally, it should be considered that, in Liverpool, a significant proportion of patients with Type IV TAAAs have undergone OSR via a thoracoabdominal approach, on left heart bypass and selective visceral perfusion, with no observed elective mortality in the past 20 years. 42 The patient population treated in this manner is significantly different than that of the present study, and we tend to reserve the thoracoabdominal approach for patients with excellent cardiorespiratory fitness. Although all our cases are discussed in a multidisciplinary setting, we do not have clearly defined indications for a thoracoabdominal approach, further complicating interpretation of our data.

## Conclusion

OSR of CAAAs carries a significant risk of early postoperative mortality and morbidity. In patients unsuitable for FEVAR or those desiring a durable long-term solution, OSR can be performed through the RP or TP approach. The RP approach facilitates treatment of more proximal CAAAs, and in certain cases allows placement of suprarenal and supramesenteric clamps, when this would not be achievable through a TP approach. It is a useful technique in the armamentarium of the modern vascular unit wanting to provide a comprehensive treatment program for patients with CAAAs, in whom more proximal clamp positions are necessary for a safe proximal anastomotic repair.
